# Periosteal incarceration versus interposition adipose tissue grafting in physeal fractures: pilot study in immature rabbits

**DOI:** 10.1186/s40634-019-0214-4

**Published:** 2019-12-01

**Authors:** Eric W. Edmonds, Joshua D. Doan, Christine L. Farnsworth

**Affiliations:** 1 Division of Orthopedic Surgery, Rady Children’s Hospital, 3020 Children’s Way, MC 5054, San Diego, CA 92123 USA; 2 Department of Orthopaedic Surgery, University of California San Diego, 3020 Children’s Way, MC 5054, San Diego, CA 92123 USA

**Keywords:** Adipose tissue graft, Periosteum incarceration, Physeal bar formation, Physeal fracture, Rabbit fracture model

## Abstract

**Purpose:**

The purpose of this study is to evaluate bar formation following physeal fracture with incarcerated periosteum or adipose tissue graft using radiographic and histological methods in an immature rabbit model.

**Methods:**

Ten-week-old rabbits underwent induced proximal tibia physeal fractures with a contralateral sham. Fractures had periosteum (*n* = 5) or adipose tissue (*n* = 5) interposed. Radiographs were compared over time by tibial medial-lateral side difference (TMLSD)(mm), femoral-tibial angle and tibia plateau angle, and physeal bars evidence. MicroCT was performed, growth plates reconstructed, and physeal area calculated and normalized to same animal contralateral physes. Physeal disruption and chondrocyte organization were evaluated histologically.

**Results:**

Radiographic: After 6 weeks, physeal bars formed in both periosteum (4 of 4) and fat groups (3 of 5). The periosteum group showed a significant increase in the TMLSD between immediate post-op and 10 days later (*p* = 0.028); but, after 6 weeks, TMLSD change was not significantly different between the three groups (*p* = 0.161). MicroCT: The normalized physeal area of every physis in the fat group was more than 0.9 (0.99 ± 0.06). Only half of the periosteum group was over 0.9 (0.81 ± 0.24). Histology: Physeal disruption was seen by microscopic evaluation in none of the sham group, all 4 in the periosteum group and 4 of 5 in the fat group.

**Conclusions:**

Fat interposition may prevent, or at least delay, the onset of bars across a fractured physis compared to periosteum, but it is not completely protective.

## Background

**Fig. 1:**
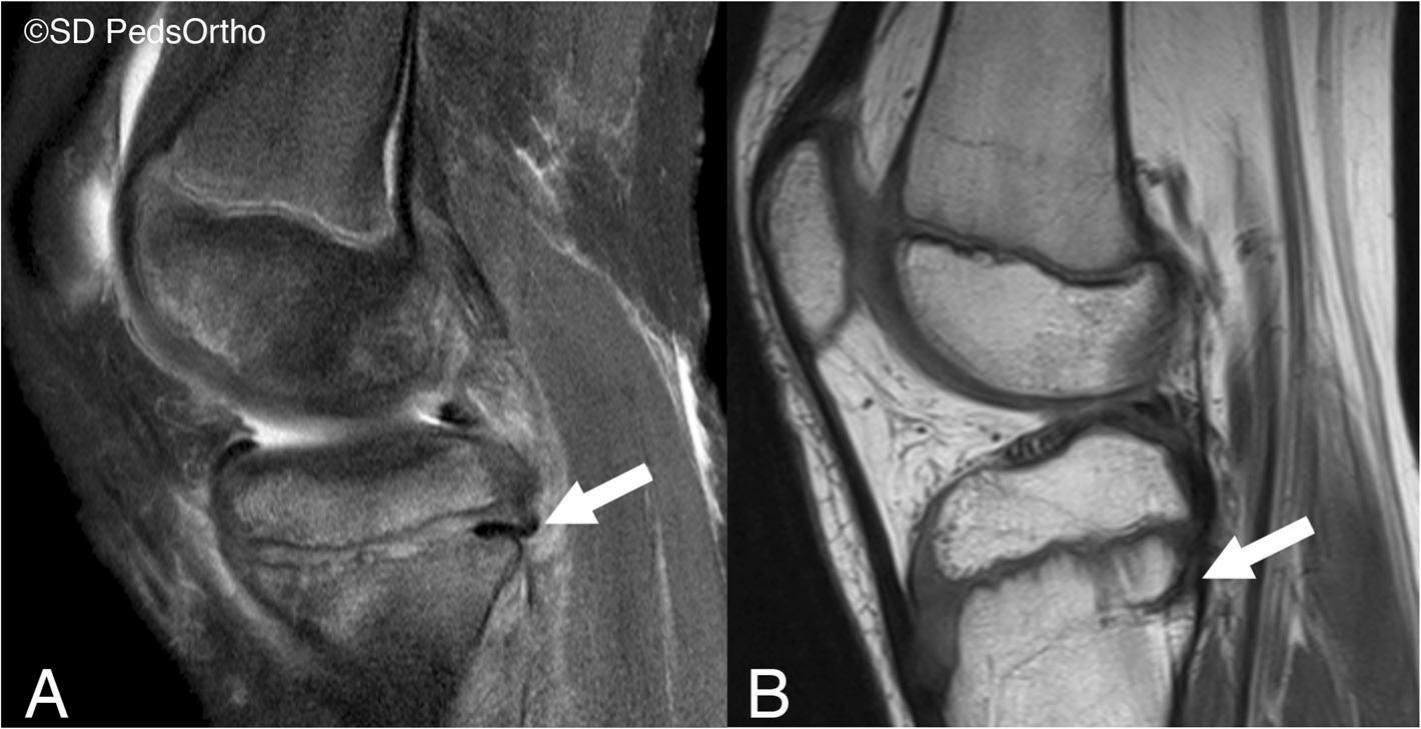
A 10 year old boy from our clinic practice. **a** Index MRI of the proximal tibial physis with evidence of periosteal tissue entrapment posteriorly (arrow); treated non-operatively. **b** 16-month follow-up MRI with continued growth, but evidence of periosteal remnant (arrow)

Approximately 15% of long bone injuries sustained during childhood involve the physis (Langenskiöld 1975; Langenskiöld et al. 1986), most have minor sequelae, but 10% end with significant growth disturbance (Salter 1994). Premature physeal closure causes growth disturbance with deformity progressing through skeletal maturity (Langenskiöld 1975; Langenskiöld et al. 1986; Vickers 1980). Previous benchwork (Gruber et al. 2002; Wattenbarger et al. 2002) utilized a rat model to demonstrate the role of incarcerated periosteum in physeal bar development, a clinical occurrence that is not rare in paediatric orthopaedics (Fig 1). Barmada et al. (Barmada et al. 2003) reported that 27% of distal tibial physeal fractures had premature physeal closure. Initial displacement, number of reduction attempts, nor treatment method affected incidence. However, physeal gaps of greater than 3mm following reduction had a 60% incidence of premature closure, compared to only 17% with no gap. Periosteum was entrapped within the physis of all gap cases, suggesting its role in premature arrest. However, a more recent study by the same institution concluded that merely removing the incarcerated periosteum during open reduction internal fixation surgery did not significantly reduce the rate of premature physeal arrest despite achieving anatomic reductions (Russo et al. 2013). Therefore, other methods of preventing a physeal arrest in these at-risk fractures are a significant need to reduce the morbidity of these injuries.

It has been proposed that replacement of portions of already damaged physes with slowly ossifying tissue may prevent premature closure (Langenskiöld et al. 1987). Lexer (Lexer 1919) first utilized free autograft adipose tissue transplantation to reduce scar tissue formation in skin healing, and orthopedic surgeons have recognized the potential merit of using free adipose tissue grafts to restore growth in the setting of known partial physeal arrests after fracture (Langenskiöld 1981; Ogden 1987; Peterson 1984; Shaw et al. 2018; Williamson and Staheli 1990). Yet, these past studies all focused on salvaging a situation wherein the previous fracture had resulted in a partial arrest and surgery was performed to remove the damaged tissue. The success of these salvage procedures have encouraged some surgeons to consider using free fat graft in the acute fracture setting with the goal of preventing the bar formation and limiting the need for future deformity correction surgery, despite the lack of controlled study and the potential risk of actually creating a physeal arrest via inducing a fracture gap (poor reduction due to interposition) or by the pluri-potential nature of the young adipose cells and pericytes (Guasti et al. 2012; Leary et al. 2009). To this end, there is a single case report of three children treated with free fat grafts in severe acute physeal fractures (Foster et al. 2000). After 2 to 5.5 years, all had open physes (x-ray), growth progression and no deformity.

Other than this single report, there is no evidence that a fat graft can further prevent physeal bar formation compared to the non-surgical option of leaving periosteum incarcerated within a physis or performing surgery to remove the incarcerated periosteum without placing an interposed graft. The rabbit proximal tibia physis has been used to study physeal fracture patterns in the proximal tibia (Jaramillo et al. 2000), providing an animal model with larger size to study regional fracture effects on the growth plate than the rat. The purpose of this pilot study was to determine if bar formation after a physeal fracture of the proximal tibia could be altered with either persistence of incarcerated periosteal tissue, or a surgically interposed adipose free graft utilizing an in vivo immature rabbit model.

## Materials and methods

Ten 10-week-old male New Zealand White rabbits (about 2 kg, all obtained from the same vendor) were used in this Institutional Animal Care and Use Committee approved pilot study. Animal care complied with the guidelines of the authors’ institution. Animals were housed in climate control rooms, individually caged, fed per the institutional animal care program feeding standard operating procedure, and given access to water ad lib.

### Surgical procedure

Rabbits were weighed then sedated via subcutaneous (SC) injection of Ketamine (35 mg/kg) and Xylazine (5 mg/kg). Anesthesia was maintained by vaporized Isoflurane (1.5–3.0% delivered via mask). Buprenorphine (0.05 mg/kg) was given SC preoperatively for analgesia. Dorsoventral (DV) and lateral radiographs were taken of both hind limbs. Animals were placed laterally recumbent and both knees shaved and sterilely prepped and draped. Surgery alternated starting on the right or left side first. A 2 cm incision was made over the lateral knee between the anterior tibialis and biceps femoris, approaching the proximal tibial growth plate. The lateral collateral ligament was protected. For the left hindlimb, the periosteum was exposed only, then the site was closed, creating the *Surgical Sham Group*.

For the right hindlimb, the periosteum was split with a scalpel in a rectangular fashion on the metaphysis and elevated from distal to proximal, creating an approximately 5 mm by 5 mm wide flap anchored on the proximal tibial epiphysis. A physeal fracture was created by either introducing an osteotome into the lateral physis and levering it to create a varus moment or via inserting two Kirschener wires (0.062 mm diameter, K-wires) into the proximal tibial epiphysis and using these to leverage against the distal tibia to create a varus moment to propagate the fracture through the physis (Salter Harris type I fracture). The medial side was kept relatively intact, as a hinge. The K-wires were removed, and the animals received one of two randomized treatments:

#### Incarcerated periosteum group

This experimental group (*n* = 5) had interposition of the locally created periosteal flap. Anchored just proximal to the perichondral ring of the physis, the periosteum was flipped into the fracture site leaving the external portion of the periosteum juxtaposed to the distal aspect of the physeal fracture. The periosteum (being only about 1 mm in thickness) was then entrapped and compressed within the physeal fracture when the gap was reduced, leaving a small residual displacement in the fracture.

#### Interposed adipose tissue (fat) group

This experimental group (*n* = 5) had interposition of an approximately 5 mm × 5 mm × 5 mm cube of free adipose tissue obtained from the retro-patellar tendon fat pad that had been exposed during the surgical approach. The cube of adipose tissue, once placed within the lateral aspect of the physeal fracture was compressed within the physeal fracture upon reduction leaving a similar slight gap in the reduction to that seen with the incarcerated periosteum strip discussed above.

All surgery was followed by absorbable suture skin closure. Dorsoventral (DV) and lateral x-rays were taken of both knees while still under anesthesia. A fiberglass cast (using 1“ rolls), with cotton lining under the cast and moleskin applied over the cast, was then applied unilaterally to the right hind limb, with an extension over the hip to allow for adequate immobilization providing rotation control. A “stirrup” shaped cutout was made over the anterior side of the foot, augmented by wooden tongue depressors, to allow dorsiflexion and assessment of the foot and ankle. Once the cast was dried, the rabbit with cast was weighed again, in order to monitor post-operative weight status of the animal, then animals were replaced in regular housing cages. Analgesia was provided postoperatively using Buprenorphine (0.03–0.05 mg/kg) delivered SC, every 6–12 h for 3 days then as needed for pain.

Ten days post-op, under repeated sedation, the casts were removed, the orthogonal radiographs were repeated, and the incision sites were assessed. Six weeks post-op, the rabbits were sedated and euthanized with an intravenous injection of pentobarbital sodium (Beuthanasia D Special, Merck Animal Health, Madison, NJ, USA). The orthogonal plain radiographs were taken of both hindlimbs which were then harvested (mid-femur to mid-tibia).

### Analysis methods

#### Radiographic methods

**Fig. 2:**
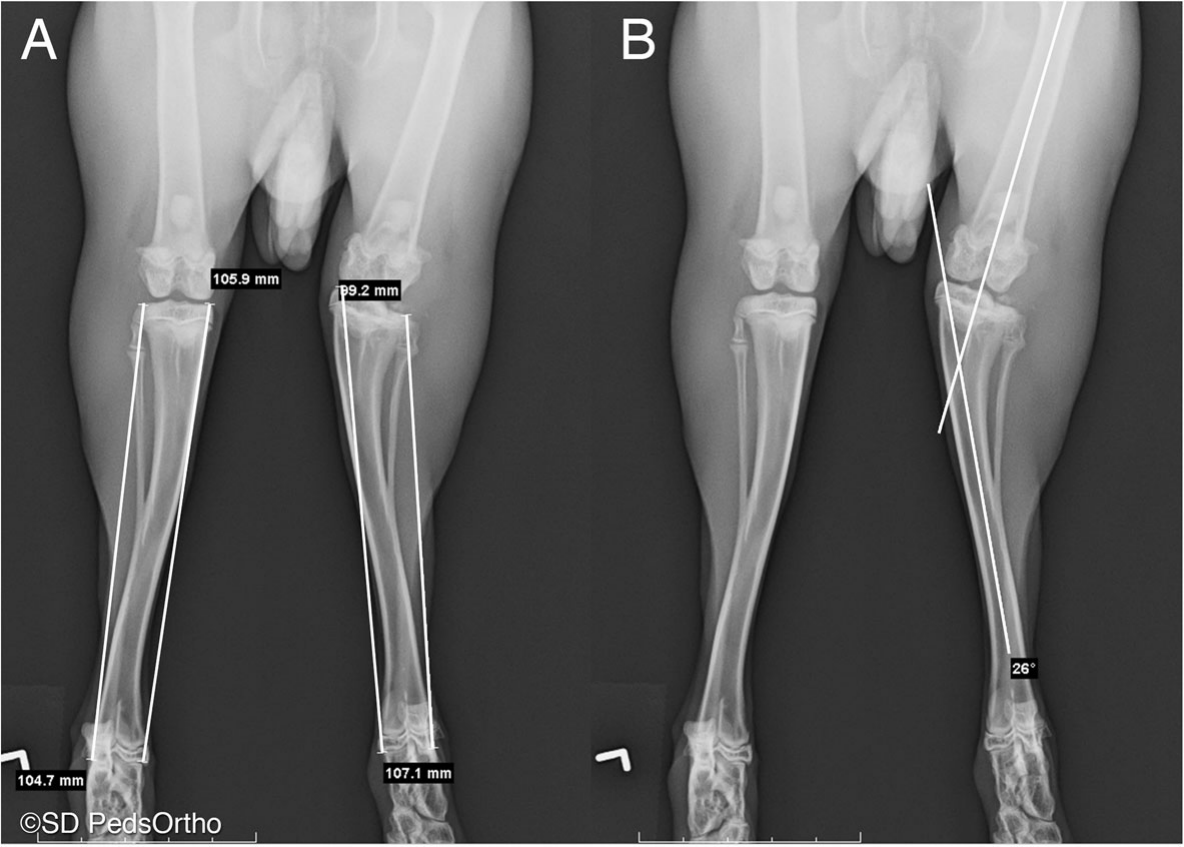
Rabbit hindlimb radiographs. **a** DV radiographs with length measurements superimposed for the medial and lateral aspect of each tibia. **b** DV radiographs with the femoral-tibial axis angle (FTA) shown

The orthogonal x-rays obtained were assessed utilizing the ruler tool provided on AMICAS PACS (AMICAS, Inc. Brighton, MA, USA). Right and left tibial lengths were measured on both the medial and lateral aspects (Fig. 2a). Angular deformity of the knee was measured using the AMICAS PACS angle tool, utilizing the longitudinal femoral axis and the tibial axis as the vectors (FTA, Fig. 2b); a valgus deformity was a positive angle and a varus deformity was a negative angle. The tibia plateau angle (PA) was also measured to further quantify the angulation of the proximal tibia. Two orthopedic surgeons independently performed all the radiographic measurements and were blinded to the treatment groups demonstrated in each radiograph.

#### Micro CT analysis

**Fig. 3:**
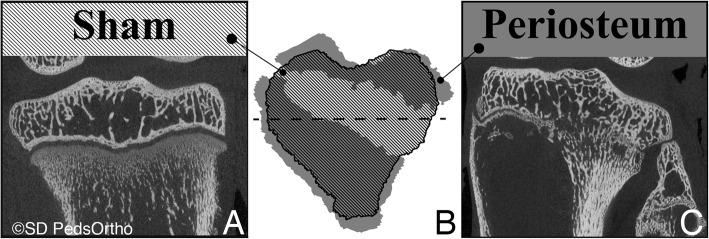
Example tibia left/Sham (**a**) and right/Periosteum (**c**) mid-coronal reconstructed microCT images. Middle image (**b**) is the schematic axial view of the right side (grey) and left (striped), superimposed and mirrored. Dashed line in B shows location of Fig. 3a and c

Micro CT (μCT) (SkyScan1076, SkyScan, Kontich, Belgium) scans were performed on freshly harvested proximal tibiae at 36 μm resolution, over 360° rotation with 0.5°step using a 1.0 aluminum filter. The images then underwent analyses that required reconstructions of individual growth plates. Physeal regions with no apparent bony bridges were traced on each coronal and sagittal μCT slice using MATLAB™ (Mathworks, Natick, MA, USA). Tracings were combined to create physeal mid-line surfaces. The surface area of each physis was calculated (Fig. 3). Surface areas of fractured physes (right side) were normalized by dividing by the surface area of the contralateral physis (sham group, left side) for each animal. Physis reconstructions with subsequent MATLAB software programming and data processing were performed by a biomedical engineer, who was blinded to the treatment groups, with extensive background with MATLAB use in orthopedic applications.

#### Histology analysis

Following μCT imaging, samples were processed for decalcified histological evaluation. All knees were snap frozen using butanol and dry ice then stored frozen at − 70 °C. They were then fixed in 10% neutral buffered formalin for 24–48 h and transferred into EDTA for decalcification. Once sufficiently decalcified, specimens were trimmed, placed into 30% sucrose, and frozen into OCT. To identify adipose tissue presence without heating the specimens which would damage the fat tissue significantly, knees were then cut in the mid-coronal plane and the surface was stained with Oil Red O (Preece 1972). High resolution digital radiographs were taken of each knee and evaluated for presence of adipose tissue within the physes. Sections were then fixed in formalin, dehydrated stepwise with ethanol, cleared with CitriSolv© (Fisherbrand, Thermo Fisher Scientific, Inc., Houston, TX, USA), embedded in paraffin blocks, sectioned at 10 μm with a microtome and fixed to glass slides. Serial mid-coronal sections were stained with hematoxylin and eosin (H&E) or Safranin O (Prophet et al. 1992), cover slipped then scanned (ScanScope, Aperio, Vista, CA, USA). Images of the slides were assessed, using a digital viewer (digital image hub v.4.0.4, Leica Biosystems Imaging Inc., Buffalo Grove, IL, USA), by a blinded researcher to determine whether the proximal tibial growth plate was disrupted, what tissue formed at the site of disruption, and the length of the disruption. Each slide was classified as ‘normal’ for no disruption in the physis, or, if present, the physeal defect was classified as ‘bone’, ‘bone/fat’, ‘fat’ or ‘cartilage’. The disruption was then measured using the given scale at each magnification. The same blinded researcher then made a qualitative analysis of the physeal cartilage organization, as compared to the Sham group physis; based on the overall columnar appearance of the physis and recorded them as: ‘organized’, or ‘unorganized’. The distinguishing criteria between these two findings being: ‘organized’, the presence of cell columns (even if leaning to the side) versus ‘unorganized’, apparent random cell placement with no evidence of columnar stacking.

#### Statistical methods

Perioperative values (age, weight, length of surgery and % weight increase) were compared between surgical groupss (Periosteum and Adipose tissue) using two sample t-tests with significance set to *p* < 0.05 (StatView version 5.0, SAS Institute Inc., Cary, NC, USA). Intra-observer correlations (ICC) were determined for each radiographic measurement (SPSS, SPSS Inc., Chicago, IL, USA). Pre-op to 6 weeks post-op x-ray measurements were compared between surgical groups (sham, periosteum, fat; Kruskal-Wallis and follow up Mann-Whitney tests, SPSS) to learn the growth effects of the surgical interventions. Radiographic measurements were also compared within each surgical group between immediate post-op and 10 days post-op to determine the effect of the post-operative treatment. μCT measures and percentage of physis closure were then compared between the surgical groups. Significance was set to *p* < 0.05.

## Results

There were no intraoperative surgical complications in this pilot study. Following successful surgery and survival period of two rabbits in each group (fat and periosteum), one rabbit in the periosteum group had major skin complications following cast removal which required early euthanization after 3.7 weeks of healing, thus 4 animals comprised the Incarcerated Periosteum group. Following this complication, the five remaining casts were modified by providing more space on the dorsal side of the foot, allowing dorsal flexion, and similar complications were subsequently avoided. There were no statistically significant differences between the rabbits in the Periosteum and Fat groups when comparing age (10.0 ± 0.7 vs. 9.8 ± 0.7 weeks, *p* = 0.651), weight (2.1 ± 0.3 vs. 2.0 ± 0.3 kg, *p* = 0.480) and weight increase (33 ± 16 vs. 39 ± 11%, *p* = 0.058) over 6 weeks. The fat group, on average, had a longer surgical duration compared to the periosteum group that did not reach significance (19 ± 10 vs 31 ± 8 min, *p* = 0.079).

### Radiographic

ICC for medial tibial length, lateral tibial length, FTA and PA were: pre-op (0.994, 0.992, 0.918, 0.683), 10 day post-op (0.985, 0.982, 0.830, 0.958) and 6 week post-op (0.663, 0.868, 0.567, 0.941). There were no significant differences in the medial to lateral tibia side difference amongst the three groups (Table 1). The periosteum group showed an increase in the tibia side difference between immediately post-op and 10 days later (*p* = 0.028), suggesting growth arrest already present after 10 days in this group. The change between pre-operative and 6 week post-op was not significantly different between the surgical groups (Sham: 0.40 ± 1.38 mm, Periosteum: 2.20 ± 2.70 mm, Fat: 4.06 ± 5.12 mm, *p* = 0.161). After completing the pilot study, a post-hoc power analysis suggests that we will need 70 animals per group in order to have 80% power to fully evaluate this measurement in future study.

**Table 1:** Radiographic Measurements

	Periosteum (*n* = 4)	Fat (*n* = 5)	Contralateral Sham
Tibia Length Side Difference (medial-lateral, mm)			
Pre-op	0.0 ± 0.9	−0.5 ± 1.3	0.5 ± 1.2
Post-op*	0.2 ± 1.3	−1.2 ± 1.5	−0.6 ± 1.7
After 10 days*	2.6 ± 1.4	1.5 ± 2.0	0.2 ± 1.2
After 6 weeks	2.2 ± 2.1	3.5 ± 3.9	0.9 ± 1.0
Femoral-Tibial Angle (FTA,°)			
Pre-op	−0.5 ± 6.0	8.6 ± 11.5	0.4 ± 8.0
Post-op	12.7 ± 9.7	3.0 ± 16.7	1.6 ± 19.0
After 10 days	18.7 ± 15.0	16.0 ± 12.3	7.3 ± 7.1
After 6 weeks	14.3 ± 6.0	18.8 ± 10.3	8.6 ± 11.1
Tibial Plateau Angle (PA, °)			
Pre-op	85.3 ± 1.5	84.2 ± 2.8	85.2 ± 4.2
Post-op	83.5 ± 2.6	87.4 ± 6.3	85.4 ± 4.6
After 10 days	76.8 ± 2.6	78.0 ± 5.9	85.3 ± 3.8
After 6 weeks	79.5 ± 5.8	71.0 ± 15.6	85.2 ± 4.1

*significant change, post-op to 10 days

The valgus alignment of the knee (femoral-tibial angle) increased slightly, not significantly, following the sham surgery at both 10 days and 6 weeks (Table 1) indicating that the rabbit knee naturally grows towards more valgus. However, both the incarcerated periosteum and the interposed fat also resulted in increased valgus angulation at the knee. The post-hoc power analysis on the pilot data indicates that only 48 animals per group are required to achieve 80% power for complete evaluation of this variable. Likewise, the tibial plateau angle remained consistent over time in the sham group; whereas, it decreased without statistical significance in the periosteum and fat surgical groups (Table 1), requiring 60 animals per group to achieve 80% power during the full comparison study.

No physeal bars were noted on any pre-operative or 10 day post-operative radiographs. After 6 weeks, tibial physeal bars were seen in each of the Incarcerated Periosteum group (4/4) and in only 3/5 of the Interposed Adipose Tissue group. Interestingly, the eliminated rabbit with periosteal interposition (only 3.7 weeks post-operative) also showed a physeal bar on radiographs (taken post-harvest).

### MicroCT

The μCT findings demonstrated that every physis in the fat group was over a ratio of 0.90 (mean of 0.99 ± 0.06) when a ratio of 1 would indicate no evidence of physeal bar and symmetric physeal surface area; whereas, only half of the periosteum group was over 0.90 (mean of 0.81 ± 0.24). Figure 3 shows an example of a periosteum group sample with some grey area that is not overlapped with stripes. In this case, the physis which had a fracture 6 weeks prior was enlarged compared to the contralateral side. This explains how the normalized fractured physeal areas were over 1.0 in some cases, despite the presence of areas of physeal closure. Periosteum and fat groups were not significantly different (*p* = 0.137); however, a power analysis demonstrated that our pilot study actually approached the desired power of 0.8, with a post-hoc power of 0.73.

### Histology

**Fig. 4:**
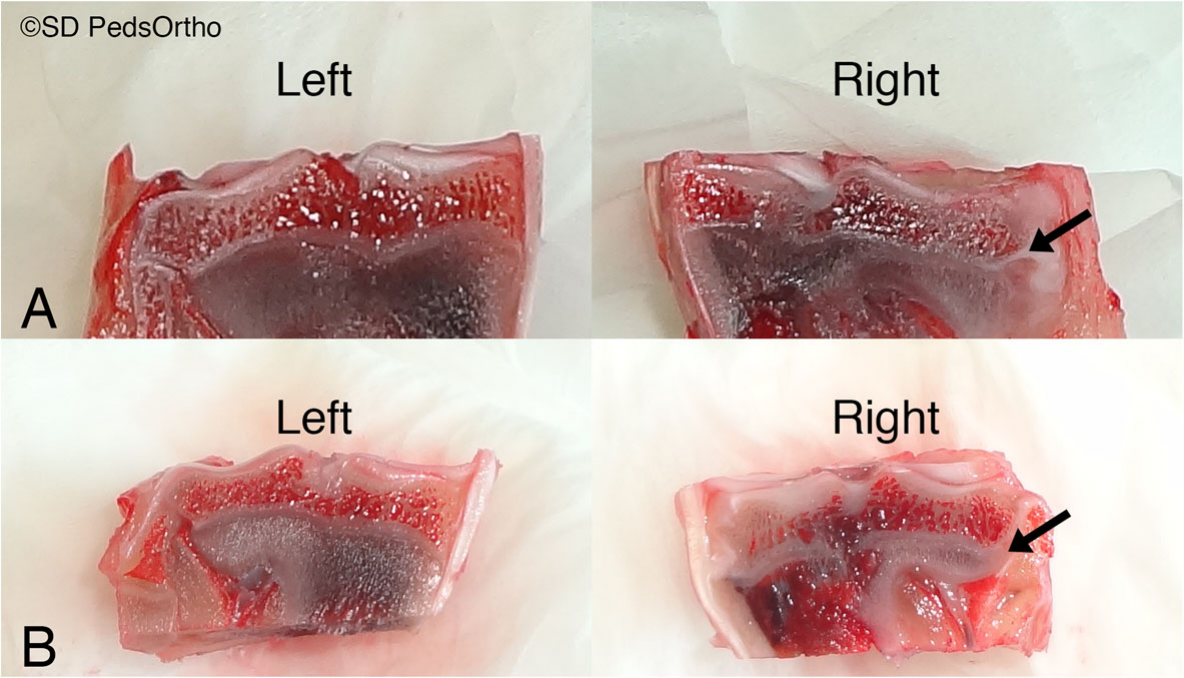
OilRedO stained mid-coronal proximal tibia section. Note, bright red stain in bone marrow indicating fat. **a** Fat Group, **b** Periosteum Group examples: arrow shows fracture and fat graft or periosteum placement location (no red stain after 6 weeks of healing)

**Fig. 5:**
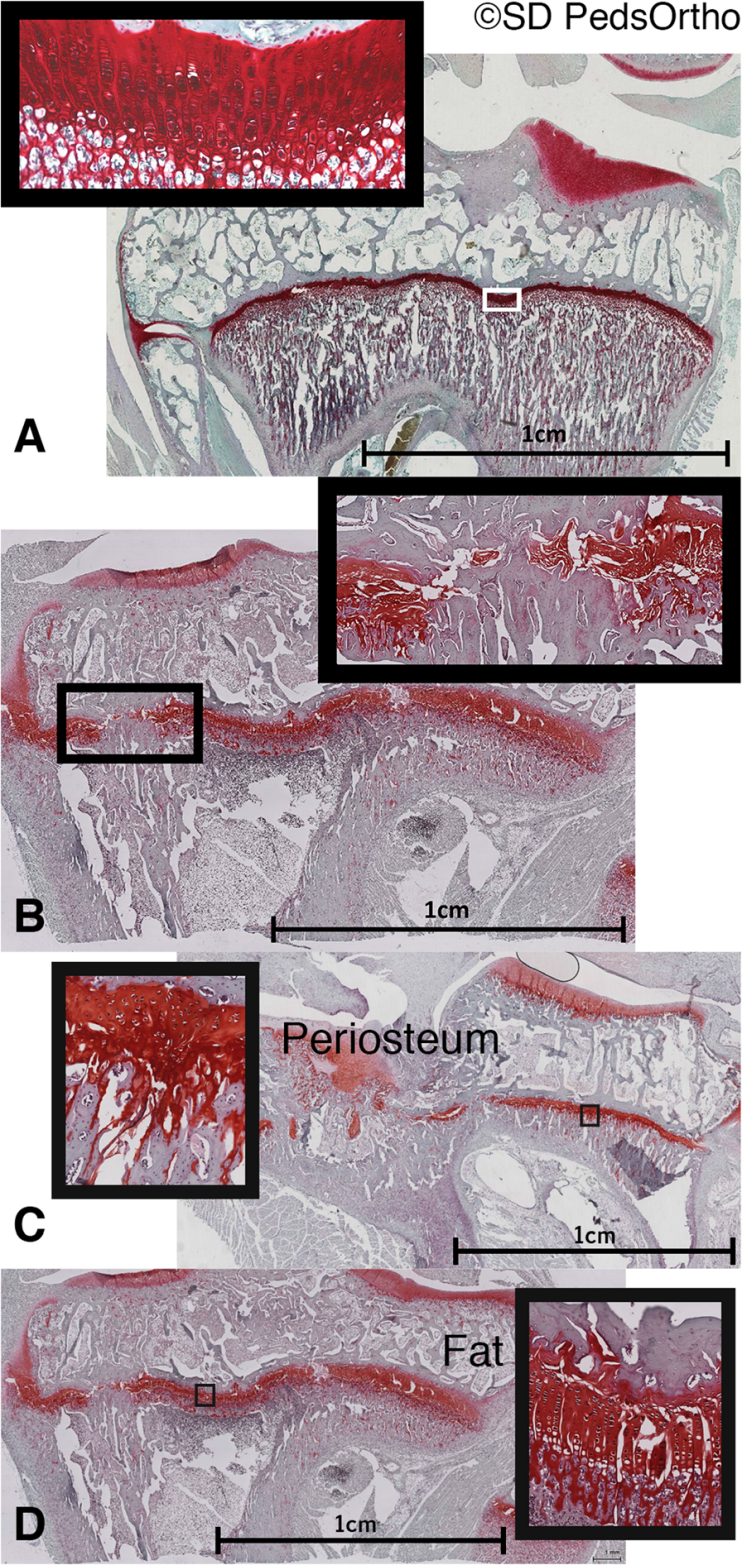
Representative mid-coronal sections (SafraninO stain). Cartilage is dark. **a** Sham Group: No bony bridges. **b** Fat Group: Bony bridge seen. **c** Representative physes from periosteum and **d** fat groups, showing disruption of chondrocyte organization compared to sham in Fig. 5a

Imaging of Oil Red O stained sections showed no macroscopic evidence of adipose cells within the fracture site (Fig. 4), suggestive of incorporation during the 6-week period. However, microscopic evaluation of the physes demonstrated slightly different findings. First, none (0/9) of the sham group, all (4/4) of the periosteum group and 4 of 5 in the fat group had disruption of the physis (Fig. 5). The periosteum group had a mean of 2.0 ± 1.8 mm disruption and Fat group had a mean 1.3 ± 1.1 mm disruption. Of note, the excluded fracture with periosteum interposition and only 3.7 weeks of healing also showed a physeal bar of 2.5 mm. Second, adipose cells were seen in 2 of 5 of the physes that received free fat grafts. There was general disruption of the chondrocytes within all growth plates of the fat and periosteum groups, which was not seen in control physes. None (0/9) of the sham group, all (4/4) of the periosteum group and 3 of 5 of the fat group showed disorganization in the remaining physeal chondrocyte organization.

## Discussion

Despite the small sample size (being only a pilot study), the μCT portion of the study (power of 73%) data suggest that pre-emptive fat interposition may at least delay the onset of bars across a fractured physis, but may not be fully protective. This appears to be consistent with clinical findings (Clark et al. 2015; Langenskiöld 1981; Ogden 1987; Peterson 1984; Sundararaj et al. 2015; Williamson and Staheli 1990) that support Lexer’s hypothesis (Lexer 1919) that a surgeon may replace the damaged portion of a growth plate with tissue that slowly undergoes ossification (such as adipose tissue) and remove a partial arrest, but it does not necessarily support the hypothesis that it might prevent a physeal arrest in the acute setting altogether. A full study, with larger sample sizes, would be necessary to draw these conclusions; however, this comparative anatomy pilot study identified two crucial points in the endeavor to reduce the morbidity of traumatic physeal arrest. First, is that a large number of rabbits would be necessary to reach statistical conclusions based on our power analysis, but this could be considered an excessive use of animals when considering the IACUC Replacement, Reduction, and Refinement mandates. Second, is that free adipose graft is not 100% effective in protecting a fractured physis from developing an arrest; and therefore, expectations need to be tempered regarding its future utilization.

Previous studies have clearly demonstrated that incarcerated periosteum plays a role in the development of physeal bars (Barmada et al. 2003; Gruber et al. 2002; Wattenbarger et al. 2002). But, the utility of surgically removing incarcerated periosteum in mildly displaced distal tibia fractures has been called into question, with evidence to suggest that surgical reduction may actually increase the risk for bar formation in itself (Chen 2015; Reynolds 1981; Russo et al. 2013). The role that interposed free adipose graft may play in physeal fracture management has not been studied in the acute setting of fracture management despite the publication of a case report (Foster et al. 2000). Yet, there is indirect evidence to support this technique of fat graft placement, if we consider the studies of excising physeal bars after they have formed and then placing the adipose grafts (Langenskiöld 1981; Ogden 1987; Peterson 1984; Williamson and Staheli 1990). Our results do not support the pre-emptive operative intervention of all physeal fractures to place adipose tissue in anticipation of a possible arrest. Yet, certain physeal fracture types (such as Salter-Harris type IV) that have an increased risk of growth disruption may benefit from interposition of adipose graft, if open reduction is already being undertaken secondary to reduction and fixation requirements (such as the aforementioned case report from Australia).

Without previous literature on the subject, the results of our pilot study need to be considered without a historical context. The plain film radiographic portion of this study was underpowered for all of the measures (a risk for Type II error – false negatives), but interestingly there was a significant difference observed at just 10 days between the two variable groups. The loss of significance in this measurement at 6 weeks either suggests that an arrest formed later in the free adipose group, or that the 6 week result is underpowered to continue a demonstration of the difference. With the other types of evaluation (microCT and histology) also indicating a physeal arrest in all groups, we believe the truth lies with delayed bar formation rather than the later issue regarding power.

A major limitation of this study is that only the fractured leg was immobilized due to IACUC concerns and restrictions, thus confounding the weight bearing of the control limb and casted fracture limb comparison. Another limitation is that the experimental group was always on the right limb and was not randomized between sides. In addition, there were no completely normal limbs for comparison, or fractured limbs without interposition of periosteum or adipose. Due to budgetary constraints, a single rabbit underwent fracture without interposition to confirm that our fracture creation technique was successful, but was not included for statistical analysis. The μCT physeal area findings are potentially confounded by apparent overgrowth of the experimental limb physis width, as compared to the control physis width. This finding allows for the control physis surface area to actually be smaller than the corresponding experimental physes – even though, the experimental physis may have a small bar present. This overgrowth was consistent for all the experimental groups and can be appreciated in the overlapping 3D image in Fig. 3, wherein the width of the experimental physis is clearly greater in all planes than the sham physis despite missing about 20% of its surface area to bar formation. Therefore, we calculated a normalized physeal area, rather than a percentage of normal; and, because each rabbit had the same overgrowth, this normalized physeal area removes the potential limitation of this surgically induced overgrowth. Overgrowth of a fractured skeletally immature bone is a known phenomenon (Reynolds 1981).

Our histologic analysis demonstrated that neither the fat nor periosteum groups had normal appearing physes at the time of final analysis. But, there were more samples with disorganization of the cartilage cells (regarding their normal columnar appearance) when adjacent to incarcerated periosteum (4 of 4) than in the fat group (3 of 5). One possible explanation may relate to the juxtaposition of these foreign tissues and a paracrine-like effect on cell differentiation. With periosteum being more closely related to bone than adipose tissue, this may cause greater disruption to the physeal chondrocytes invoking changes that can form a physeal bar. This effect is yet to be elucidated fully and is merely speculative. Future study to evaluate this will require the evaluation of stem cells within different tissue types to determine the rate of conversion of cell type given the physeal fracture milieu of inhibitors and catalyst for growth.

This study is limited in that the rabbit model only mimics the complicated clinical situation where there is a growth plate fracture exacerbated by the presence of periosteum within the fracture site. However, short of conducting a clinical prospective randomized trial, the animal model is a tried and true manner to compare two clinically relevant treatments. Another possible limitation of this pilot study is the utilization of the rabbit model over the traditional rodent mode (Gruber et al. 2002; Wattenbarger et al. 2002). The rabbit physis is larger than the rodent physes, facilitating the procedure of interposing fat into the fractured physis whilst maintaining a good reduction of the fracture, and we did achieve physeal bar formation. The other main limitation, at least in regards to drawing final conclusions, is that this was only a pilot study and most variables evaluated were under-powered, with only one measure being powered at 73%. The results of the pilot study, however underpowered, still suggest a very pertinent finding: physeal bar formation is still possible even with a free adipose graft interposed in the physeal fracture.

Risks of placing a free adipose graft need to be considered, as well. Although there is risk for donor site morbidity not identified in this pilot study, this is a potential area for complication. Our results did demonstrate evidence for extended operative time in the free adipose tissue group. Finally, there may be a risk for inadequate fracture reduction due to the interposition of the adipose graft that could result in mal-alignment, although none of the hindlimbs grew into varus due to a poor reduction of the fracture. In fact, they grew into valgus, but the amount of deformity is under-represented as 78% of our rabbits developed a physeal arrest that would likely have resulted in further deformity should they have continued growth to maturity.

## Conclusions

The null hypothesis of this study was that we would find no difference between the surgical groups, and although there is a difference seen when looking at the number and size of physeal bars that formed between the groups, the fact remains that both groups could form a physeal bar. Although the study is underpowered for the other radiographic measurements, the μCT findings suggest that pre-emptive interposition of a free adipose graft improves the rate of physeal bar formation over leaving incarcerated periosteum, and warrants further clinical study. Because this is already being performed in human children to presumably protect the physis, future clinical study can utilize MRI sequencing to evaluate the long-term effect at the physis through prospective evaluation, as the size of bar, size of physis and duration of remaining growth may all play a role in the ultimate success of free adipose grafting in physeal fractures. Without this above information from future study, then the unfortunate conclusion from this pilot study is that the risk of physeal bar formation persists even when physeal fractures are treated at the index surgery utilizing the time-honoured belief that a free adipose graft interposition could stop the formation.

## Data Availability

The datasets used and/or analysed during the current study are available from the corresponding author on reasonable request.
